# Deciphering the biology of KIR2DL3^+^ T lymphocytes that are associated to relapse in haploidentical HSCT

**DOI:** 10.1038/s41598-021-95245-7

**Published:** 2021-08-04

**Authors:** Gaëlle David, Catherine Willem, Nolwenn Legrand, Zakia Djaoud, Pierre Mérieau, Alexandre Walencik, Thierry Guillaume, Katia Gagne, Patrice Chevallier, Christelle Retière

**Affiliations:** 1 Etablissement Français du Sang-Pays de la Loire, Blood Bank, 34 boulevard Jean Monnet, 44011 Nantes Cedex 01, France; 2 CRCINA, INSERM, CNRS, Université d’Angers, Université de Nantes, 44000 Nantes, France; 3 LabEx IGO “Immunotherapy, Graft, Oncology”, 44000 Nantes, France; 4 LabEx Transplantex, Université de Strasbourg, 67000 Strasbourg, France; 5 Hematology Clinic, CHU, 44000 Nantes, France

**Keywords:** Cancer, Immunology

## Abstract

KIR are mainly expressed on NK cells and to a lesser extent on T lymphocytes. Although the KIR NK cell repertoire was well explored in haploidentical Hematopoietic Stem Cell Transplantation (HSCT), KIR T cell compartment remains to be investigated in this context. In this study, the investigation of NK receptors on T lymphocytes during immune reconstitution after T-cell-replete haploidentical HSCT with Post-Transplant Cyclophosphamide (PTCy) has shown a significant increase of KIR2DL2/3^+^ T cell frequency at day 25. This was especially observed at day 30 in recipients who relapsed. IL-15 but not IL-12 increased in vitro KIR^+^ T cell expansion suggesting that the raised IL-15 serum concentration observed after PTCy in haploidentical HSCT might increase KIR^+^ T cell frequency. Moreover, investigations from healthy blood donors showed a higher inhibiting effect of KIR2DL3 on CMV specific T cell response against allogeneic than autologous C1^+^ target cells. The association of KIR^+^ T cell subset with relapse may suggest that inhibitory KIR2DL2/3 limit anti-leukemic effect of specific T lymphocytes at this early step of immune reconstitution. Further phenotypic and mechanistic investigations on this cell subset from a broader cohort of HSCT recipients should clarify its potential implication in relapse occurrence. Our results demonstrate that KIR-HLA interactions known to modulate NK cell functions also modulate T cell immune responses in the context of allogeneic HSCT.

## Introduction

The Immunoglobulin superfamily clusters numerous major molecules implicated in T and NK cellular immunity as CD3, CD4, CD8, HLA class I, HLA class II molecules and KIR (Killer cell Immunoglobulin like Receptors). These latter are mainly expressed on Natural Killer (NK) cells and constitute key receptors engaged with HLA class I molecules to modulate NK cell responses. The KIR gene family comprises 16 genes located on chromosome 19. KIR2DL1, KIR2DL2 and KIR2DL3 are the most described receptors that recognize HLA-C molecules depending on the nature of amino acid at position 80 and leads to 2 groups of ligands : C1 (Asn80) and C2 (Lys80). Thus, KIR2DL1 recognizes HLA-C molecules of C2 group and KIR2DL2 and KIR2DL3 which are allelic genes recognize HLA-C molecules of C1 group^1^. However, with others^2–4^, we reported a larger spectrum of HLA-C recognition by KIR2DL2 and KIR2DL3 than KIR2DL1 which specifically reacted against C2 allotypes^5,6^. KIR3DL1 recognizes some HLA-A and HLA-B molecules sharing Bw4 sequence motifs^7^.

NK cells sense the absence of autologous HLA class I molecules via inhibitory KIR. This mechanism called “missing self” recognition^8^ is particularly important in allogeneic context in which autologous HLA molecules are absent and also on virally infected cells or tumoral cells in which HLA class I expression can be down-regulated. However, KIR are not specific NK markers and they are also expressed on αβ^9,10^ and γδ T lymphocytes^11^. KIR are acquired by conventional αβ T lymphocytes after the termination of T cell receptor rearrangements^12–14^ at the transition step between effector to memory status^15^. The KIR^+^ T lymphocytes are mainly CD8^+^ CD27^−^ CD28^−^ and they express CD45RA and CD57 but they do not express CCR7^16^. KIR expression was documented on CD4^+^ T lymphocytes in CMV seropositive individuals^17^. The frequency of KIR^+^ memory T cells raises with increasing age^18^. It has been shown in mouse model that KIR confer a better capacity of T lymphocytes to proliferate and survive^19^. One particular case is represented by KIR3DL2 which is expressed on clonal T lymphocyte population in Sézary syndrome^20^ and constitutes a specific cell surface marker for the follow-up of patients^21^. The minor CD56^+^ T lymphocyte subset that share common phenotypic markers with NK cells, express KIR and KIR^+^ CD56^+^ T cell frequency can drastically increase with CMV infection^22^.

The KIR gene regulation and expression differ between T and NK cells^16^. Whereas all KIR genes in NK cells are transcripted and translated to be expressed, they are regulated at both pre and posttranscriptional levels on T lymphocytes explaining in part the low diversity of KIR expression on T cells^13^. KIR expression is induced by CpG DNA demethylation in CD8^+^ T lymphocytes suggesting an epigenetic regulation of KIR on T lymphocytes^23^. The inhibition of the DNA methyltransferase (DNMT) maintains a global KIR expression in NK cells. However, DNMT plays its complete role in T lymphocytes inhibiting KIR expression. The decreased DNMT levels observed with increasing age in CD8^+^ T lymphocytes contribute to KIR expression that dampen immune T cell responses in the elderly.

HLA molecules take a major place in immunology as modulators of both innate and adaptive cellular immunities. HLA class I molecules are allele specifically recognized by KIR expressed mainly on Natural Killer (NK) cells. The recognition of self-HLA molecules by KIR contribute to NK cell education^24^. The strength of the KIR/HLA interaction during NK cell development determines the threshold of NK cell activation. Thus, only KIR NK cell subsets “licensed” to sense the presence of self-HLA class I ligands are able to sense their absence. Thus, KIR/HLA interactions inhibit NK cell functions in physiological conditions and down-regulation or absence of HLA class I membrane expression triggers KIR NK cell functions in pathological or allogeneic contexts. In contrast, peptide-loaded HLA molecules specifically trigger T lymphocytes via TcR recognition. In addition, KIR expressed on T lymphocytes can inhibit T cell activation mediated by TcR-HLA recognition. It therefore remains to establish whether HLA class I environment modulates KIR^+^ T cell function and the potential consequences in HLA haploidentical HSCT. In this study, we investigated KIR expression on T lymphocytes during immune reconstitution after haploidentical HSCT with PTCy from a cohort previously described and studied for NK cell reconstitution^25^. In parallel, to decipher the KIR^+^ T cell biology, we firstly investigated the impact of cytokines on their expansion and we secondly evaluated the impact of allogeneic HLA environment on KIR modulation of T lymphocyte function using an in vitro model based on HLA-A2-pp65 specific T lymphocytes.

## Results

### Relapse is associated with higher KIR2DL2/3^+^ T cell frequency around GvHD median time point after T-replete haploidentical HSCT with PTCy

**Figure 1:**
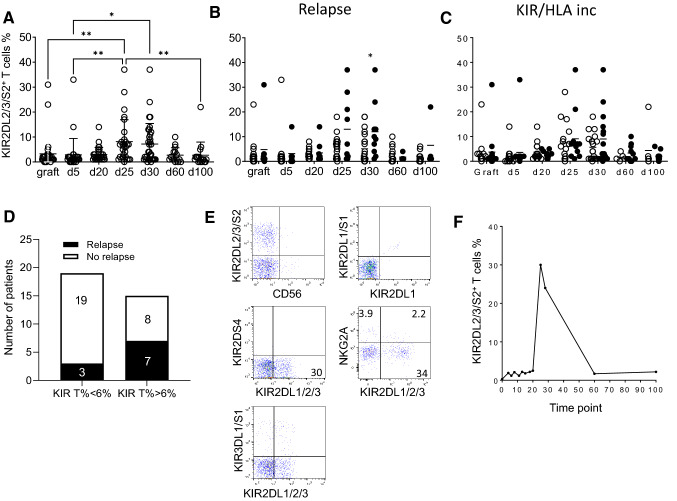
Increased frequency of KIR2DL2/3/2DS2^+^ T lymphocytes at days 25–30 associated with relapse occurrence. **(A)** KIR2DL2/3/2DS2^+^ T cell frequencies determined by flow cytometry from grafts and recipients (n = 34) after T-replete haploidentical HSCT with PTCy, following the post transplantation kinetic (days 5, 20, 25, 30, 60 and 100), **(B)** according to relapse occurrence (white circle for no relapse and black circle for relapse) and **(C)** inh. KIR/HLA inc. (white circle for no inc. and black circle for inc.). Comparisons of KIR2DL2/3^+^ T cell frequencies (mean rank) at different time points were performed using a nonparametric test (Kruskal–Wallis test) with no matching or pairing. Comparisons of KIR2DL2/3^+^ T cell frequencies between patients with relapse or not were performed by multiple Mann–Whitney tests of two-way-ANOVA using the method of two-stage step-up (Benjamini, Krieger, and Yekutieli) * and **indicate *p* < 0.05 and *p* < 0.001 respectively. **(D)** A contingency analysis was performed using Chi-square test to compare patients with relapse or not in groups with less and more than 6% of KIR2DL2/3^+^ T cells. **(E)** Density plots illustrating the T cell phenotype at day 25 for a representative patient. **(F)** Kinetic of KIR2DL2/3/2DS2^+^ T cell appearance determined by flow cytometry from a representative recipient following post-transplantation time points (days 0, 4, 6, 8, 11, 13, 15, 18, 20, 25, 28, 60 and 100).

In a recent publication, we have reported the beneficial impact of KIR/HLA incompatibilities on patient outcome after T-replete haploidentical HSCT with PTCy^25^. In parallel to NK cell compartment, we investigated T lymphocyte reconstitution. For 33 donor/recipient pairs, we investigated NK receptor expression (NKp30, NKp44, NKp46, NKG2D, DNAM-1, 2B4, NKG2A, NKG2C, KIR2DL1/S1, KIR3DL1, KIR3DS1 and KIR2DS4) on T lymphocytes (data not shown). Among all monitored data, we observed an increased frequency of KIR2DL2/3/S2^+^ T cells during immune reconstitution after HSCT, particularly at day 25 compared to graft (0.007) and day 5 (0.001) and at day 30 compared to day 5 (0.02) (Fig. 1A). Interestingly, patients with relapse had a higher KIR2DL2/3/S2^+^ T cell frequency at day 30 (mean rank of no relapse 14.61% vs mean rank of relapse 22.50%, p = 0.02), around GvHD median time point (29 days) (Fig. 1B). For the unique KIR2DL2^+^/KIR2DS2^+^ genotyped donors, 1F12/GL183 antibody combination led us to identify KIR2DL2 expression in accordance with a preferential inhibitory KIR expression on T cells (Suppl. Fig. S1). Of note, neither KIR/HLA incompatibilities (Fig. 1C), nor GvHD or treatment (data not shown) had significant association with KIR2DL2/3/S2^+^ T cell frequency. No association was observed with KIR or HLA genetics of donors and recipients (data not shown). A patient’s stratification based on KIR2DL2/3 expression has been done. The threshold of 6% of KIR2DL2/3^+^ T cells has been defined to separate patients with low and high KIR2DL2/3^+^ T cell frequency. Thus the representation of relapsed patients was significantly higher (p = 0.04) in the group with high KIR2DL2/3^+^ T cell frequency (Fig. 1D). All donors and recipients for whom a high KIR2DL2/3^+^ T cell frequency is observed were C1^+^ (6 C1C1 and 1 C1C2). In contrast, no C2C2 patients (n = 5) harbored KIR^+^ T cell expansion. This increased frequency of KIR^+^ T lymphocytes focused only on KIR2DL2/3 and not on KIR2DL1, KIR3DS1 or KIR2DS4 as documented for a representative recipient at day 25 in Fig. 1E. Moreover, the KIR2DL2/3^+^ T lymphocytes are preferentially NKG2A^−^. Interestingly, the peak of KIR2DL2/3^+^ T cell frequency was observed only between day 25 and day 30 during immune reconstitution as represented for a representative recipient with relapse (Fig. 1F). Of note, at this stage, the proportion of CD56^+^ T lymphocytes was very low and CD3^+^ T lymphocytes were massively CD56^−^ (data not shown). It is conceivable that inhibitory KIR2DL2/3 limit anti-leukemic effect of specific T lymphocytes at this step of immune reconstitution contributing to relapse occurrence.

### IL-15 stimulates KIR^+^ lymphocyte expansion

**Figure 2:**
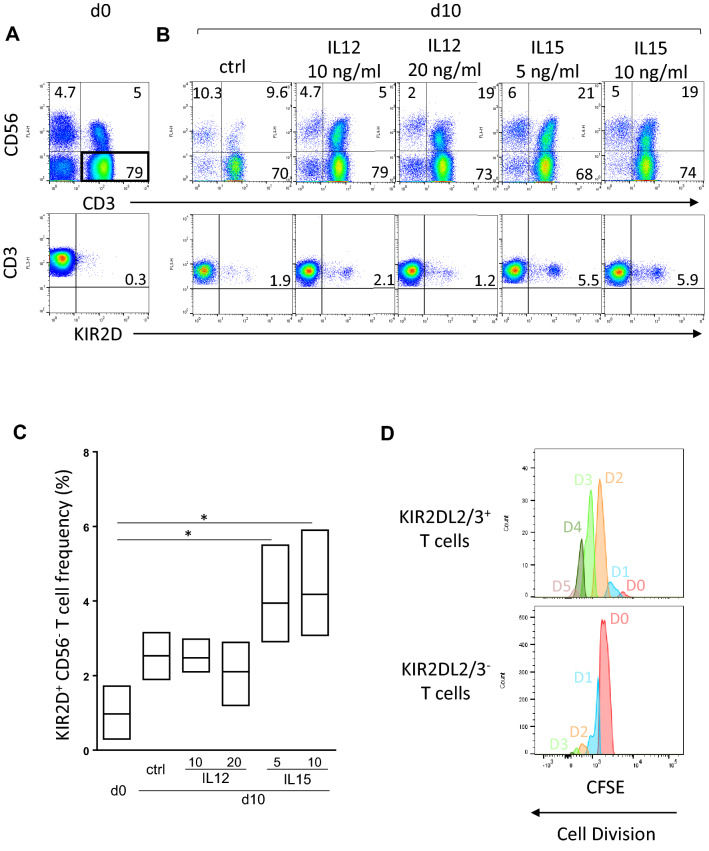
IL-15 stimulates KIR^+^ lymphocyte expansion. **(A)** Representative density plots illustrating KIR2DL1/2/3/2DS1/2 expression on CD56^−^ T cell subsets targeted using CD3 and CD56 combination at day 0 by flow cytometry **(B)** and day 10 after PBMC culture with IL-2 at 50 U/ml alone or in combination with IL-12 (10 and 20 ng/ml) or IL-15 (5 and 10 ng/ml). The cell frequency is indicated on all density plots. **(C)** Floating bars (min to max) compiling results of different experiments realized from 3 healthy blood donors. *Indicates *p* < 0.05, ANOVA. **(D)** Representative histograms of KIR^−^ and KIR^+^ T cell proliferation by the method of CFSE that allows the identification of dividing cells stimulated for 6 days with 10 ng/ml IL-15. Generation’s gates are indicated (DO to D5).

In haploidentical HSCT, a raised IL-15 serum concentration has been documented early after the cyclophosphamide administration^26^. To evaluate the impact of cytokines on KIR^+^ (KIR2DL1/S1 and KIR2DL2/3/2DS2) CD56^−^ T cell frequency, we evaluated IL-2 alone or in combination with IL-12 or IL-15, known to modulate NK receptor expression on T lymphocytes in focusing on CD56^−^ T lymphocytes whose frequency increased between day 25 and day 30 during immune reconstitution (Fig. 2A). Interestingly, we observed after 10 days of culture that only IL-15 at 5 or 10 ng/ml in combination with IL-2 increased the KIR^+^ CD56^−^ T cell frequency as illustrated for a representative blood donor (Fig. 2B) and confirmed on 3 blood donors (Fig. 2C). However, as the increased frequency of KIR^+^ NK cells after cytokine stimulation can be due to a privileged loss of KIR^−^ T lymphocytes or induction of KIR expression of KIR^−^ T cells, we assessed the effect of 6 days IL-15 stimulation on the proliferation of KIR^−^ and KIR^+^ T cell subsets using CFSE marker (Fig. 2D). Thus, our results show that IL-15 stimulates more KIR^+^ than KIR^−^ T lymphocyte proliferation. Thus, it is conceivable that IL-15 enriched HSCT context favors increased KIR^+^ CD56^−^ T cell frequency in the first month after HSCT.

### Lower ex vivo degranulation of CMV specific KIR^+^ T lymphocytes compared to KIR^−^ counterpart in HSCT recipients

**Figure 3:**
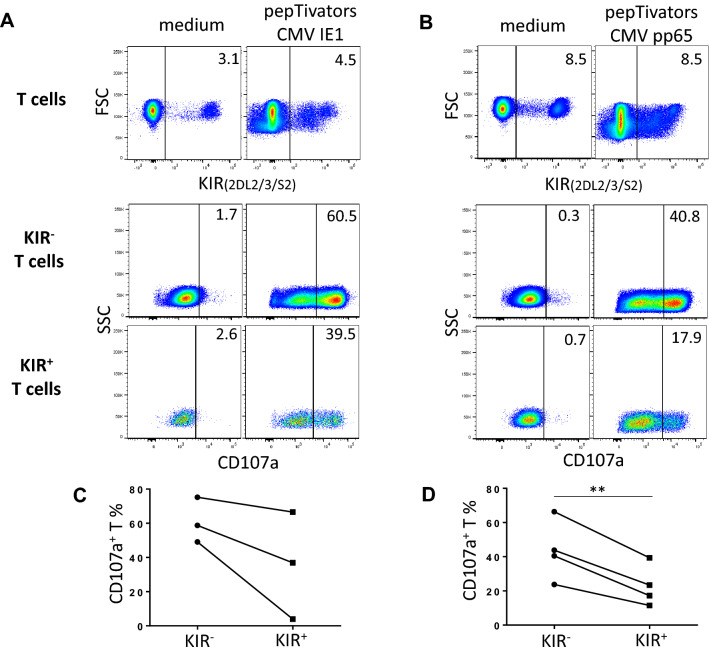
Lower ex vivo degranulation of CMV specific KIR^+^ T lymphocytes compared to KIR^−^ counterpart in HSCT recipients. Density plots illustrating the ex vivo degranulation of **(A)** IE1 and **(B)** pp65 specific KIR2DL2/3^−^ and KIR2DL2/3^+^ T lymphocytes from HSCT recipients. PBMC at day 100 post-graft were incubated with IE1 or pp65 peptivators during 6 h. T cell degranulation observed in 5 recipients against CMV with 3 specific responses against IE1 **(C)** and 4 specific responses against pp65 **(D)**. Student’s *t* test. **Indicates *p* < 0.01.

**Figure 4:**
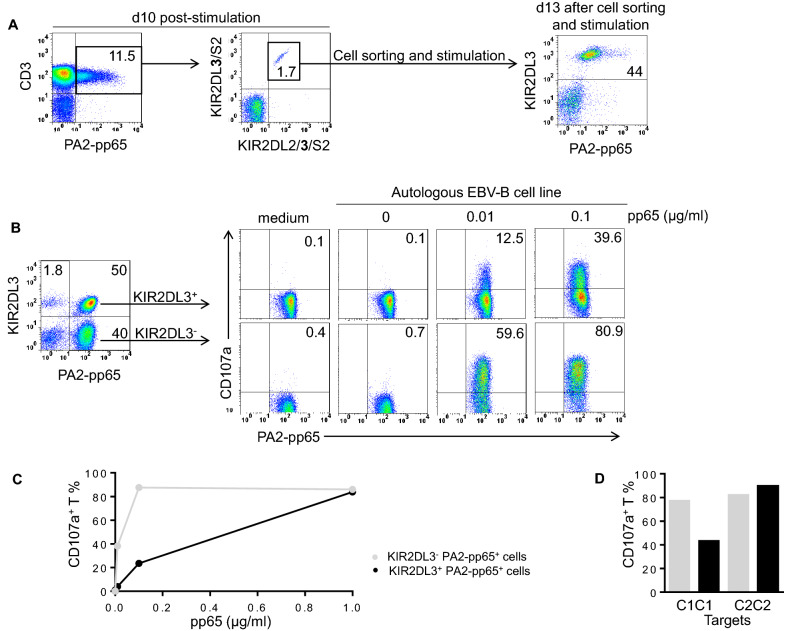
KIR2DL3 inhibits the degranulation of HLA-A2-pp65 specific T lymphocytes. **(A)** PBMC from HLA-A2^+^ CMV^+^ individuals were stimulated using pp65 loaded autologous PBMC following the ratio E: T of 1:1. After 10 days of stimulation, HLA-A2-pp65 specific T lymphocytes were targeted using specific pentamer (PA2-pp65) in combination with KIR2DL2/3/S2 specific mAb (GL183) and KIR2DS2/KIR2DL3 specific mAb (1F12). KIR2DL3^+^ PA2-pp65^+^ lymphocytes were cell sorted and in vitro amplified by stimulation as illustrating for one representative individual (I10) of 3. **(B)** After day 15, the frequency of PA2-pp65 increased and simultaneously, KIR2DL3^+^ HLA-A2-pp65 specific lymphocytes frequency decreased leading to investigate in parallel the function of KIR2DL3^−^ and KIR2DL3^+^ HLA-A2-pp65 specific lymphocytes. Density plots of KIR2DL3^+^ and KIR2DL3^−^ HLA-A2-pp65 specific T cell degranulation observed after 5 h incubation alone (medium) or in the presence of pp65 (0, 0.01 and 0.1 µg/ml) loaded autologous EBV-B cell line, at an effector: target ratio of 10:1 for one representative individual (I10) of 3. **(C)** KIR2DL3^+^ and KIR2DL3^−^ HLA-A2-pp65 specific T cell degranulation observed after 5 h incubation alone (medium) or in the presence of pp65 (0, 0.01, 0.1 and 1 µg/ml) loaded autologous EBV-B cell line, at an effector: target ratio of 10:1 for one representative individual (I1) of 5. (D) KIR2DL3^+^ and KIR2DL3^−^ HLA-A2-pp65 specific T cell degranulation observed after 5 h incubation in the presence of pp65 (0.01 µg/ml) loaded C1C1 EBV-B cell line (HiD) or C2C2 autologous EBV-B cell lines, at an effector: target ratio of 10:1 for one representative individual (I19) of 5.

We further evaluated whether KIR2DL2/3^+^ T lymphocytes are less efficient than KIR^−^ T lymphocytes in HSCT recipients. As it is difficult to investigate a leukemic specific T cell response from different patients using a well-characterized model, we focused our study on CMV specific T cell responses that it is large, comprising as much as 10–20% of all CD8^+^ αβ T cells^27,28^. We evaluated the ex vivo degranulation of IE1 and pp65 specific KIR2DL2/3^−^ and KIR2DL2/3^+^ T lymphocytes from HSCT recipients at day 100 post-graft with a CMV profile (D^+^/R^+^, D^+^/R^+^ and D^−^/R^+^) as illustrated for one recipient (Fig. 3A,B). T cell degranulation was observed in 5 recipients against CMV with 3 specific responses against IE1 (Fig. 3C) and 4 specific responses against pp65 (Fig. 3D). The CMV specific degranulation was lower for KIR2DL2/3^+^ T lymphocytes than KIR2DL2/3^−^ counterparts and significantly lower using pp65 peptivators. Of note, all these recipients express C1, KIR2DL2/3 ligands. Our results suggest that KIR2DL2/3^+^ expression on T lymphocytes confers a lower degranulation potential than KIR2DL2/3^−^ counterparts during the immune reconstitution.

### Expression of HLA specific receptor on HLA-A2-pp65 specific T lymphocytes used as cellular model to investigate allogeneic HLA class I impacts on KIR T cell functions

To further evaluate how KIR2DL3 can modulate T cell function in autologous and allogeneic contexts, we focused our investigation on HLA-A2-pp65 specific T lymphocytes. From a broad cohort of 200 blood donors, we selected 31 CMV^+^ HLA-A2^+^ donors. HLA-A2-pp65 specific lymphocytes have been observed using pentamer (PA2-pp65) staining (Fig. 4A) or after pp65 specific in vitro amplification. Thus, we identified HLA-A2-pp65 specific T lymphocytes from 21 CMV^+^ HLA-A2^+^ donors (Table 1). Based on KIR genotyping, we identified 6 C1C1, 10 C1C2 and 5 C2C2 CMV^+^ HLA-A2^+^ KIR2DL2/3^+^ individuals. We amplified KIR2DL3^+^ HLA-A2-pp65 specific T lymphocytes from these different individuals (Table 1) through stimulation with autologous PBMC loaded with pp65. After 10 days amplification of HLA-A2-pp65 specific T lymphocytes, KIR2DL3^+^ T cells were cell sorted with respect to KIR2DL3 and PA2-pp65 and in vitro amplified to enrich the polyclonal KIR2DL3^+^ HLA-A2-pp65 specific T lymphocytes as illustrated for a representative C1C2 donor in Fig. 4A. After day 15, the frequency of PA2-pp65 increased and simultaneously, KIR2DL3^+^ HLA-A2-pp65 specific lymphocytes frequency decreased leading to investigate in parallel the function of KIR2DL3^−^ and KIR2DL3^+^ HLA-A2-pp65 specific lymphocytes (Fig. 4B). The phenotype of enriched KIR2DL3^+^ HLA-A2-pp65 specific lymphocytes was checked to ensure the absence of other KIR expression (KIR2DL1/S1, KIR2DL2, KIR3DL1/S1). Of note, KIR2DL3^−^ and KIR2DL3^+^ HLA-A2-pp65 specific lymphocytes presented similar phenotype (NKG2C^−^, NKG2A^−^, CD16^−^, ILT2^−^, NKp46^−^, DNAM-1^+^ and CD56^−^) (data not shown). As shown in Fig. 4B, the degranulation of KIR2DL3^+^ HLA-A2-pp65 specific T lymphocytes (C1C1 I1) is lower than those of KIR2DL3^−^ counterpart, suggesting an inhibitory effect of KIR2DL3 on specific T lymphocyte activation. However, a high concentration of peptide (1 µg/ml) bypassed the inhibitory effect of KIR2DL3 leading to obtain the plateau of the dose–response curve (Fig. 4C) as both KIR2DL3^−^ and KIR2DL3^+^ HLA-A2-pp65 specific T cell subsets have a similar cytolytic potential against C1 deficient target cells (Fig. 4D).

**Table 1:** HLA and KIR genotypes of studied CMV^+^ volunteer blood donors.

Donor	HLA typing						KIR ligands	KIR typing															KIR genotype
	A	A	B	B	C	C		2DL1	2DL2	2DL3	2DL4	2DL5	3DL1	3DL2	3DL3	2DS1	2DS2	2DS3	2DS4	1D	2DS5	3DS1	
I1	02:01	03:01	18:01	44:03	12:03	16:01	Bw6Bw4 C1C1	+	−	+	+	−	+	+	+	−	−	−	−	+	−	−	AA
I2	02:01	33:01	52:01	58:01	07:01	12:02	Bw4Bw4 C1C1	+	−	+	+	−	+	+	+	−	−	−	−	+	−	−	AA
I3	01:01	02:01	08:01	39:06	07:01	07:02	Bw6Bw6 C1C1	+	+	+	+	−	+	+	+	−	+	−	+	+	−	−	B +
I4	02:01	33:01	14:02	15:01	03	08	Bw6Bw6 C1C1	−	+	−	+	+	+	+	+	+	+	−	−	+	+	+	B +
I5	02:01	02:01	15:01	40:01	03:04	03:04	Bw6Bw6 C1C1	+	+	+	+	+	+	+	+	+	+	+	−	+	+	+	B +
I6	02:01	11:01	08:01	14:02	07:01	08:02	Bw6Bw6 C1C1	+	−	+	+	+	+	+	+	+	−	+	−	+	+	+	B +
I7	02:01	02:01	51:01	57:01	06:02	07:02	Bw4Bw6 C1C2	+	−	+	+	−	+	+	+	−	−	−	−	+	−	−	AA
I8	02:01	03:01	35:03	40:01	03:04	04:01	Bw6Bw6 C1C2	+	−	+	+	−	+	+	+	−	−	−	−	+	−	−	AA
I9	02:01	11:01	18:01	44:02	05:01	12:03	Bw4Bw6 C1C2	+	−	+	+	−	+	+	+	−	−	−	−	+	−	−	AA
I10	02:01	66:01	40:01	51:01	03:04	16:02	Bw4Bw6 C1C2	+	−	+	+	−	+	+	+	−	−	−	+	+	−	−	AA
I11	02:01	03:01	15:01	49:01	04:01	07:01	Bw4Bw6 C1C2	+	+	+	+	−	+	+	+	−	+	−	+	−	−	−	B +
I12	02:01	24:02	18:01	40:02	02:02	07:01	Bw6Bw6 C1C2	+	+	+	+	+	+	+	+	+	+	+	−	+	+	+	B +
I13	02:01	02:01	35:08	44:03	04:01	16:01	Bw4Bw6 C1C2	+	+	+	+	+	+	+	+	+	+	+	+	+	+	+	B +
I14	02:01	03:01	08:01	35:01	04:01	07:01	Bw6Bw6 C1C2	+	+	+	+	+	+	+	+	+	+	+	−	+	−	+	B +
I15	02	68	18	51	07	15	Bw4Bw6 C1C2	+	+	+	+	+	+	+	+	+	+	−	+	−	+	+	B +
I16	02	03	51:01	55:01	03	15	Bw4Bw6 C1C2	+	+	+	+	+	+	+	+	+	+	−	+	−	+	+	B +
I17	02:01	24:02	13:02	44:02	05:01	06:02	Bw4Bw4 C2C2	+	+	−	+	+	+	+	+	−	+	+	+	+	−	−	B +
I18	01:01	02:01	07:05	57:03	06:02	15:05	Bw4Bw6 C2C2	−	+	−	+	−	+	+	+	−	+	−	+	+	−	−	B +
I19	02:01	26:01	18:01	40:02	02:02	05:01	Bw6Bw6 C2C2	+	−	+	+	+	+	+	+	+	−	−	−	+	+	+	B +
I20	02	24	35	57	04	06	Bw4Bw6 C2C2	+	+	−	+	+	+	+	+	−	+	+	−	+	−	−	B +
I21	02	31	44	44	02	05	Bw4Bw4 C2C2	+	−	+	+	−	+	+	+	−	+	−	+	+	−	−	B +

### KIR2DL3 inhibits T lymphocyte degranulation against autologous target cells only in C1^+^ individuals

**Figure 5:**
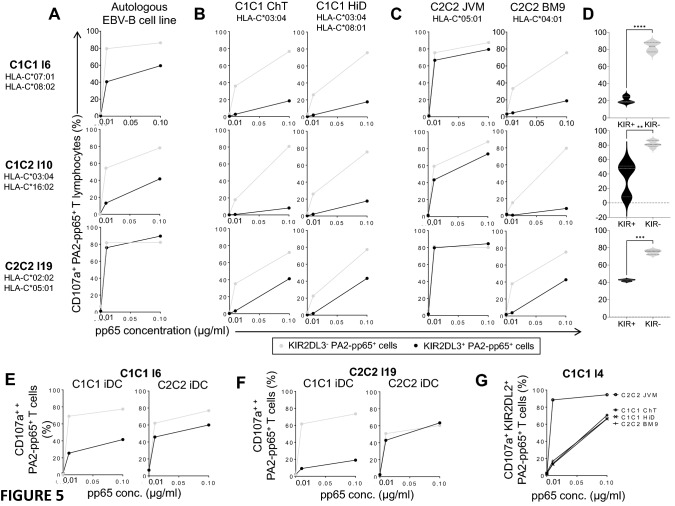
KIR2DL3 inhibits conventional T lymphocyte response against target cells expressing C1 ligands and HLA-C*04 C2 ligand with higher effect on allogeneic than autologous ligands. **(A)** KIR2DL3^+^ and KIR2DL3^−^ HLA-A2-pp65 specific T cell degranulation observed after 5 h incubation in the presence of pp65 (0, 0.01, and 0.1 µg/ml) loaded autologous EBV-B cell line, **(B)** allogeneic C1C1 ChT (HLA-C*03:04) and HiD (HLA-C*03:04; C*08:01) EBV-B cell lines, **(C)** C2C2 JVM (HLA-C*05:01) and BM9 (HLA-C*04:01) EBV-B cell lines at an effector: target ratio of 10:1 for three representative individuals (C1C1 I6, C1C2 I10 and C2C2 I19). Experiments were performed from 5 C1C1, 3 C1C2 and 2 C2C2 blood donors. **(D)** Violin plots of KIR2DL3^+^ and KIR2DL3^−^ HLA-A2-pp65 specific T cell degranulation observed after 5 h incubation in the presence of pp65 (0.1 µg/ml) loaded C1C1 EBV-B cell lines for 5 C1C1, 3 C1C2 and 2 C2C2 blood donors. Paired t test was used to evaluate whether values between KIR2DL3^+^ and KIR2DL3^−^ T cell degranulation were significantly different. **, *** and ****indicate *p* < 0.01, *p* < 0.001 and *p* < 0.0001 respectively. **(E)** KIR2DL3^+^ and KIR2DL3^−^ HLA-A2-pp65 specific T cell degranulation observed after 5 h incubation in the presence of pp65 (0, 0.01, and 0.1 µg/ml) loaded allogeneic C1C1 and C2C2 immature dendritic cells at an effector: target ratio of 10:1 for one representative C1C1 (I6) and **(F)** C2C2 individuals (I19). **(G)** KIR2DL2^+^ HLA-A2-pp65 specific T cell degranulation observed after 5 h incubation in the presence of pp65 (0, 0.01, and 0.1 µg/ml) loaded allogeneic C1C1 (ChT, HiD) and C2C2 (JVM, BM9) EBV-B cell lines for one representative C1C1 individual (I4).

Based on the model of the functional education on KIR^+^ NK cells by self-HLA class I molecules, we raised the question of the nature of autologous HLA-Cw environment on the modulation of T lymphocyte function by KIR. To reply to this question, we studied the degranulation of different KIR2DL3^+^ HLA-A2-pp65 specific T lymphocytes enriched from C1^+^ (n = 5) and C1^−^ (n = 2) individuals against pp65 loaded autologous EBV-B cell line. KIR2DL3^+^ HLA-A2-pp65 specific T lymphocytes from one representative C1C1 individual and C1C2 individual were less activated by C1^+^ autologous EBV-B cell line than their KIR2DL3^−^ counterparts (Fig. 5A). However, KIR2DL3^+^ HLA-A2-pp65 specific T lymphocytes from one representative C2C2 individual present a similar degranulation than KIR2DL3^−^ counterpart against autologous target cells (Fig. 5A) that can be explained by the low affinity of KIR2DL3 for C2 ligands as HLA-C*02:02, HLA-C*05:01 encoded molecules. Thus, in autologous context, KIR2DL3 inhibits T lymphocyte functions only in C1^+^ individuals.

### Impact of allogeneic HLA environment on modulation of CMV-specific T lymphocyte function by KIR2DL3

In allogeneic HSCT, KIR expressed on T lymphocytes can be engaged with allogeneic HLA-Cw molecules and modulate T cell function. We, therefore, evaluated the degranulation of KIR2DL3^−^ and KIR2DL3^+^ HLA-A2-pp65 specific T lymphocytes enriched from C1C1 I6, C1C2 I10 and C2C2 I19 individuals against different HLA-A2^+^ allogeneic EBV-B cell lines as target cells co-expressing different HLA-C molecules: C1C1 ChT (HLA-C*03:04), C1C1 HiD (HLA-C*03:04, *08:01), C2C2 JVM (HLA-C*05:01) and C2C2 BM9 (HLA-C*04:01). KIR2DL3 inhibited the degranulation of HLA-A2-pp65 specific T lymphocytes against all C1^+^ target cells (Fig. 5B) for all individuals whatever their HLA-C environment. Interestingly for C1^+^ individuals, the degranulation of KIR2DL3^−^ and KIR2DL3^+^ HLA-A2-pp65 specific T lymphocytes was higher with autologous EBV-B cell line in contrast to studied C1^+^ allogeneic EBV-B cell lines. KIR2DL3 inhibition was more efficient in allogeneic environment than autologous one. However, KIR2DL3 did not inhibit the degranulation of HLA-A2-pp65 specific T lymphocytes against C2C2 JVM EBV-B cell line which expresses HLA-C*05:01 encoded molecule (Fig. 5C). In contrast, C2C2 BM9 EBV-B cell line which expressed HLA-C*04:01 encoded molecule inhibited strongly KIR2DL3^+^ HLA-A2-pp65 specific T lymphocyte degranulation as all C1^+^ target cells for C1^−^ and C1^+^ individuals (Fig. 5A–C) as summarized in Fig. 5D for all studied C1C1 (p < 0.0001), C1C2 (p = 0.002) and C2C2 (p = 0.0007) blood donors. This result is in accordance with our previous study on KIR2DL3^+^ NK cell recognition showing that HLA-C*04:01 encoded molecule grouped as C2 ligand was better recognized than some C1 ligands. These observations were also shown using allogeneic C1C1 and C2C2 immature dendritic cells (iDC) for C1C1 (Fig. 5E) and C2C2 individuals (Fig. 5F). As KIR2DL3, KIR2DL2 inhibited HLA-A2-pp65 specific T cell degranulation from a representative C1C1 individual against C1^+^ and HLA-C*04^+^ BM9 target cells but not C2C2 JVM target cells (Fig. 5G). This inhibition profile of KIR2DL2 was confirmed for C2C2 individual (data not shown). Interestingly, the inhibitory effect of KIR2DL2 was bypassed by the peptide concentration as of 0.1 µg/ml (Fig. 5G). Thus, these results show that KIR2DL3 inhibits conventional T lymphocyte response against target cells expressing HLA-C molecules from C1 group and HLA-C*04 C2 ligand recognized as C1 with higher effect on allogeneic than autologous ligands.

### KIR2DL3 expression on T lymphocytes inhibits their apoptosis

**Figure 6:**
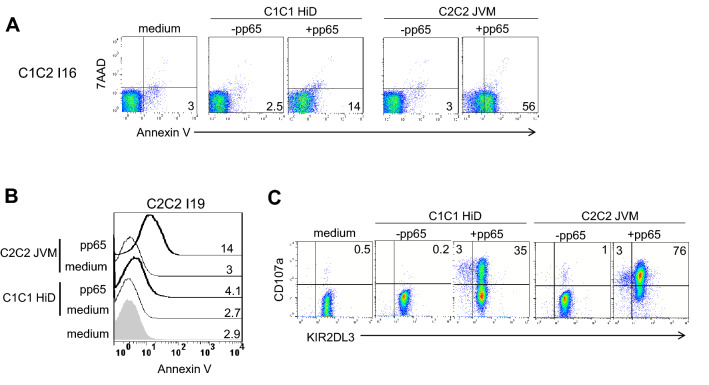
KIR2DL3 inhibits T lymphocyte apoptosis. **(A)** Density plots illustrating apoptotic HLA-A2-pp65 specific KIR2DL2/3^+^ T lymphocytes as annexin V^+^ 7AAD^−^ after 3 h incubation alone or in presence of pp65 (0.1 µg/ml) loaded allogeneic C1C1 (ChT, HiD) and C2C2 (JVM, BM9) EBV-B cell lines for one representative C1C2 individual (I16) performed twice and for 2 C1C2 individuals. Mean Fluorescent Intensity (MFI) of Annexin V^+^ cells is indicated on all density plots. **(B)** Histograms representing annexin V expression by HLA-A2-pp65 specific KIR2DL3^+^ T lymphocytes after 3 h incubation alone or in presence of pp65 (0 and 0.1 µg/ml) loaded allogeneic C1C1 (ChT, HiD) and C2C2 (JVM, BM9) EBV-B cell lines for one C2C2 individual (I19). Mean Fluorescent Intensity (MFI) of Annexin V^+^ cells is indicated for all conditions. **(C)** Density plots illustrating HLA-A2-pp65 specific KIR2DL3^+^ T cell degranulation in the presence of pp65 (0 and 0.1 µg/ml) loaded allogeneic C1C1 (ChT, HiD) and C2C2 (JVM, BM9) EBV-B cell lines for one C2C2 individual (I19).

Using KIR-HLA transgenic mice, it has been shown that recognition of HLA class I molecules by KIR on T lymphocytes down-regulates activation-induced cell death promoting their survival^19^. In this study, we revisited the question of KIR impact on T cell apoptosis using our model based on KIR2DL3^+^ HLA-A2-pp65 specific T lymphocytes. In presence of pp65 loaded allogeneic C1C1 HLA-A2^+^ EBV cell line, the frequency of apoptotic lymphocytes was low in contrast to the condition using pp65 loaded allogeneic C2C2 HLA-A2^+^ EBV cell line in which pp65 specific T lymphocytes were activated (Fig. 6A). These observations were also made from a representative C2C2 I19 individual (Fig. 6B). Apoptotic lymphocytes were more important after activation with pp65 loaded C2C2 compared to C1C1 target cells in accordance with the degranulation of KIR2DL3^+^ HLA-A2-pp65 specific T lymphocytes in these stimulation conditions (Fig. 6C). Thus, KIR2DL3 inhibits specific T cell activation against C1^+^ target cells and decreases their apoptosis.

## Discussion

In this study, we reported an increased frequency of KIR2DL2/3^+^ T lymphocytes in the predominant CD56^−^ cell compartment during immune reconstitution after T-replete haploidentical HSCT with PTCy with a significant increase around day 25. Interestingly, we observed that this higher frequency of KIR2DL2/3^+^ T lymphocytes is significant at day 30 in recipients who relapsed. Our results are consistent with a recent study of Noviello et al. showing that bone marrow central memory and memory stem T cells express inhibitory receptors implicated in their exhaustion in AML patients relapsing after allogeneic HSCT^29^. The tumor-specific T cells are exhausted early after HSCT. Analysis of activation markers on T lymphocytes around day 30 post-HSCT showed a high activation of T lymphocytes as observed by increased CD69 frequency and T lymphocytes concomitantly expressed the inhibitory marker, PD-1, indicative of recent TCR signaling and/or exhaustion^30^. Moreover, some phenotypic perturbations detected in bone marrow T cells are also observed in the peripheral blood. Nonetheless, little is known about the environmental factors implicated in the expansion of KIR^+^ T lymphocytes. Expansion of KIR^+^ CMV-specific T lymphocytes has been reported in allogeneic HSCT in patients receiving T-cell depleted graft with post-graft CMV reactivation^31^. The authors have suggested that strong antigen specific stimulation could trigger CMV-specific T cell amplification leading the detection of scarcely represented KIR^+^ T cells. In our study, no association was observed with CMV infection or reactivation in patients. KIR^+^ T cells express the common β chain of the interleukin 2 and interleukin 15 receptors. In addition, IL-15 and its receptor play an important role in generation and maintenance of memory CD8^+^ T cells^32^. As IL-15 is produced during immune reconstitution after haploidentical HSCT, it is possible that the cytokine environment favors the enrichment of allogeneic KIR^+^ T lymphocytes. Indeed, cytokines as IL-15 can be implicated in the induction of KIR^+^ T lymphocytes as previously described for CD94/NKG2A^+^ T lymphocytes^33,34^ and CD56^+^ T lymphocytes^35^. In our study, only IL-15 increases preferentially the expansion of KIR^+^ T lymphocytes. Interestingly, CD56^+^ T lymphocytes which share NKR with NK cells were also particularly expanded in vitro with IL-15 at 5 n/ml (data not shown) as previously reported^35^. Nonetheless, this population was not observed during the immune reconstitution of studied HSCT patients and KIR expression was focused on CD56^−^ T lymphocytes. Following IL-15 stimulation, STAT5 is activated and may modulate DNMT^32,36^ that result in demethylation of DNA and potentially KIR expression. Treatment with DNA methylase inhibitor of cord blood T lymphocytes can induce KIR expression^37^. In addition, in accordance with our observations, KIR expression can be induced on cord blood T lymphocytes after in vitro IL-15 stimulation for 2 weeks.

Recently, Gimeno et al. have shown that KIR2DL1^+^ CD8^+^ T cells showed a gene expression signature related to efficient tumor immunosurveillance, whereas KIR2DL2/L3^+^ CD8^+^ T cells showed transcriptomic profiles related to suppressive anti-tumor responses^38^. In our study, only an increased frequency of KIR2DL2/3^+^ T cells was observed in recipients but no difference was observed for KIR2DL1^+^ T lymphocytes. Moreover, we have shown that CMV specific KIR2DL2/3^+^ T cells in recipients at day 100 harbor a lower degranulation potential than KIR2DL2/3^−^ T cells. In this study, we did not evaluate KIR2DL3 expression on CD4 and CD8 T lymphocytes; nevertheless KIR2DL3^+^ T lymphocytes observed in high frequency at day 30 are likely CD8^+^ as KIRs are preferentially expressed on effector memory CD8^+^ T lymphocytes^39^. KIR expression is coordinated with the acquisition of cytotoxic function in human CD8^+^ T cells^40^. In T-replete haploidentical HSCT with PTCY, proliferative T lymphocytes are early eliminated with PTCY^26^ but memory stem T cells (T_SCM)_ accumulate. The latter are preferentially generated from naive precursors in vivo early after haploidentical HSCT when homeostatic cytokines as IL-7 and IL-15 are particularly abundant^41^. Donor derived T_SCM_ are highly enriched after HSCT and an active T lymphocyte proliferation starts off from day 15^41^. Overall, our data support that expanded KIR2DL2/3^+^ T cells observed early during immune reconstitution may contribute to relapse. Therefore, broader cohorts and further phenotypic and functional investigations are needed to evaluate the direct implication of these expanded KIR2DL2/3^+^ T cells in limiting anti-leukemia effect.

To document the impact of allogeneic HLA class I molecules on the modulation of KIR2DL3^+^ T cell function and in absence of well-established leukemia model, we focused our investigations on HLA-A2-pp65 specific T lymphocytes. Indeed, KIR expression has been previously documented on these cells in allogeneic HSCT patients^31,42^. HLA-C*04:01 encoded molecule belongs to C2 group of KIR ligands. However, we have reported, with other groups^3–6^ that this HLA-C ligand is recognized as C1 ligand by KIR2DL3 expressed on NK cells. Interestingly in our study, we observed a strong inhibition of KIR2DL3^+^ T lymphocytes activated with homozygous HLA-C*04:01 BM9 cell line confirming the recognition of HLA-C*04 molecules by KIR2DL3 as C1 ligand on T lymphocytes. Interestingly, both previous studies based on KIR2DL3^+^ T cell clones have used only homozygous HLA-C*04:01 BM9 cell line as C1^−^ target cells that can explain the low inhibiting effect of KIR2DL3 in their assay^13,31^.

We have shown that KIR2DL3 engagement with HLA C1 ligands decreases activation and apoptosis of T lymphocytes. These observations are consistent with previous report showing that KIR expression on memory CD8^+^ T cells confers better capacity to proliferate and survive^19^. The specific activation of KIR^+^ T lymphocytes by HLA-peptide complex on allogeneic target cells in absence of KIR ligand triggers probably T cell functions and the cell death by apoptosis. Thus, the prompt apparition of KIR^+^ T lymphocytes during immune reconstitution between day 25 and day 30 suggests an in vivo activation of these KIR^+^ T lymphocytes followed by their disappearance.

In conclusion, the high frequency of KIR^+^ T lymphocytes observed after in vitro culture with IL-15 strongly supports that early IL-15 production in haploidentical HSCT^26^ can favor the enrichment of this T cell subset. The early anti-leukemic response in the first month is important. In that respect, the expression of HLA specific inhibitory receptor as KIR on T lymphocytes at this post-graft step might likely weaken the cellular immune responses and the clinical outcome of patients. Further phenotypic and mechanistic investigations on early-expanded KIR^+^ T lymphocytes from a broader cohort of HSCT recipients should clarify their potential implication in relapse occurrence.

## Methods

### Study population

This prospective study, conducted between November 2013 and May 2017, enrolled 33 patients (Table 2). The source of graft was PBSC for all cases. This cohort has been recently described^25^. This biologic study was approved by the Ethic Review Board of the Nantes University Hospital and all patients and donors included provided informed consent for collecting their own data from the PROMISE database of the EBMT.

**Table 2:** Characteristics and outcome of patients.

Gender: male	22 (67%)
Median age: years (range)	60 (32–70)
**Disease**	
Myeloid/lymphoid	24 (73%)/9 (27%)
**Status at transplant**	
CR1/CR2/CR3	16/2/1 (58%)
PR1/PR2/PR3	2/0/3 (15%)
Active	8 (24%)
**Disease risk index**	
Low	4 (12%)
Intermediate	20 (61%)
High	9 (27%)
Previous allograft	4 (12%)
**Conditioning**	
Baltimore	11 (33%)
Clo-Baltimore	22 (67%)
**Haplo-donors**	
Median age	42 (22–71)
Sister/brother	5/9 (42%)
Father/mother	2/1 (9%)
Son/daughter	10/3 (39%)
Nephew	3 (9%)
**Donor/recipient CMV status**	
−/−	21 (64%)
−/+	3 (9%)
+/−	5 (15%)
+/+	4 (12%)
**ABO compatibility**	
Compatibility	24 (73%)
Minor inc	6 (18%)
Major inc	3 (9%)
**Graft composition**	
Median CD34 + cells: 10 × 6/kg	7.88 (2.88–14.16)
Median CD3 + T cells: 10 × 7/kg	22.69 (7.71–38.71)
Median CD45 + cells: 10 × 8/kg	7.9 (5.04–16.06)
Follow-up: months (range)	29.1 (12.4–50)
Median neutrophils recovery > 0.5 Giga/L: days (range)	18 (13–30)
Median platelets recovery > 50 Giga/L: days (range)	30.5 (14–104)
**Acute GVHD**	19 (58%)
Grade 2	14 (42%)
Grade 3	2 (6%)
Grade 4	3 (9%)
Median time of ocurence: days (range)	29 (5.7–121)
Relapse	11 (33%)
2-year DFS	50.3% (95% CI 38–66)
2-year OS	61.6.0% (95% CI 49–76)
Deaths	13 (39%)
**Causes of death**	
Relapse	7 (54%)
Infection	1 (8%)
GHVD	4 (31%)
Dermatomyositis	1 (8%)

*CR* complete remission, *PR* partial remission, *CMV* cytomegalovirus.

### Cells (PBMC and cell lines)

PBMC were isolated as previously described^43^. All blood donors were recruited at the Blood Transfusion Center (EFS, Nantes, France) and informed consent was given by all donors. A declaration of the preparation and conservation of a bio-collection (DC-2014-2340) was submitted to the French Ministry of Research and had received agreement from the IRB (2015-DC-1) .HLA-A2^+^ allogeneic EBV-B cell lines from the tenth International Histocompatibility Workshop (IHW) panel^44,45^ or from blood donors were used as target cells to evaluate T lymphocyte degranulation: C1C1 ChT (HLA-C*03:04), C1C1 HiD (IHW9074, HLA-C*03:04, *08:01), C2C2 JVM (IHW9039, HLA-C*05:01) and C2C2 BM9 (IHW9068, HLA-C*04:01). Autologous B-cell lines were established by EBV transformation of peripheral B cells using EBV supernatant harvested from the cell line B95-8 (American Type Culture Collection). Immature dendritic cells (iDC) were obtained from C1C1 and C2C2 blood volunteer donors as previously described^46^.

### HLA and KIR genotyping

HLA class I allele assignment and KIR gene content typing were performed as previously described^25^ for all blood volunteer donors. KIR generic typing was performed on all donor samples using a KIR multiplex PCR-SSP method as previously described^47^ using primers provided by Dr Ketevan Gendzekhadze (City of Hope, Duarte, CA, USA).

### CFSE staining

T cell proliferation was determined by carboxyfluorescein diacetate succinimidyl ester (CFSE) method (Molecular Probes, Eugene, OR, USA). Previously thawed PBMC were labeled with a 5 μM CFSE in PBS (1 nM final concentration) with 2% FCS for 6 min at 37 °C. The labeling reaction was quenched by addition of cold RPMI 1640 with 10% FCS, and cells were washed twice with PBS with 2% FCS to remove excess CFSE. Labeled PBMC were cultured in the presence of 10 ng/ml IL-15 at 37 °C and 5% CO_2,_ for six days. The analysis was performed using the proliferation platform of Flowjo™ 10.2 software (LLC, Ashland, OR, USA).

### Selection and amplification of KIR2DL2/3^+^ HLA-A2-pp65 specific T lymphocytes

Thawed PBMC from CMV^+^ HLA-A2^+^ blood donors were divided in 2 parts. The first one was pulsed with pp65 at a concentration of 1 µg/ml for 1 h before washing and used to stimulate the second part of autologous PBMC in a 1:1 ratio (stimulator: effector). The cells were cultured in RPMI 1640 medium (Gibco, Paisley, Scotland, UK) containing glutamine (Gibco) and penicillin–streptomycin (Gibco) supplemented with 10% human serum (EFS, Nantes) and 50 U/ml of IL-2 (Chiron, Suresnes, France). HLA-A2-pp65 specific T lymphocytes were amplified in 10 days. Then, KIR2DL3^+^ HLA-A2-pp65 specific T lymphocytes were cell sorted (FACSCalibur, BD Biosciences, San Jose, CA, USA) using the combination of KIR2DL3/2DS2-FITC (1F12^48^), HLA-A2-pp65 pentamer-PE (ProImmune), CD3-PerCP (SK7, BD Biosciences) and KIR2DL2/3/2DS2-APC (GL183, Beckman Coulter, La Brea, CA, USA) mAbs. Cell sorted KIR2DL2/3^+^ HLA-A2-pp65 specific T lymphocytes were stimulated with irradiated allogeneic feeders and amplified 2 weeks before functional analysis.

### Phenotypic analysis by flow cytometry

The T cell surface phenotype was determined by flow cytometry using the following mouse anti-human mAbs: anti-KIR2DL1-FITC (143211, R&D Systems), anti-KIR2DL1/2DS1-PE (EB6, Beckman Coulter), anti-KIR2DL2/3/2DS2-PE (GL183, Beckman Coulter), anti-CD3-PerCP (SK7, BD Biosciences), anti-CD56-APC (B159, BD Biosciences), anti-KIR2DL3/2DS2 (1F12, EFS, Nantes, France), anti-KIR3DL1/3DS1 (Z27, Beckman Coulter), anti-KIR2DS4 (FES172, Beckman Coulter) and anti-NKG2A (Z199, Beckman Coulter). For cytokine stimulation, PBMC were cultivated with IL-2 at 50 U/ml alone or in combination with IL-12 (R&D systems) (10 and 20 ng/ml) or IL-15 (R&D systems) (5 and 10 ng/ml).

### CD107a mobilization assay and apoptosis detected by flow cytometry

The Peptivators CMV IE1 and pp65 (Milteniy Biotec, Bergisch Gladbach, Allemagne) were used to stimulate 6 h T cell degranulation following manufacture’s recommandations. Enriched KIR2DL3^+^ HLA-A2-pp65 specific T lymphocytes were pre-incubated with anti-CD107a (H4A3, BD Biosciences). T-cell degranulation was assessed after incubation for 5 h alone (negative control), or with different target cells (E:T ratio = 10:1) with brefeldin A (Sigma-Aldrich, Saint-Louis, MO, USA) at 10 μg/ml for the last 4 h. Cell surface staining was performed using the anti-human KIR 1F12 and GL183 mAbs and HLA-2-pp65 specific pentamer. To evaluate apoptosis in T lymphocytes, cells were incubated 3 h at 37 °C with target cells using a ratio 10:1 (E:T) before the staining with 7-AAD and Annexin-V (BD Biosciences). All flow cytometry data were collected using a FACSCalibur (BD Biosciences) and analyzed with Flowjo™ 10.2 software (LLC, Ashland, OR, USA).

### Statistical analyses

Comparisons of KIR2DL2/3^+^ T cell frequencies (mean rank) at different time points were performed using a nonparametric test (Kruskal–Wallis test) with no matching or pairing. Comparisons of KIR2DL2/3^+^ T cell frequencies (mean rank) between patients with relapse or not were performed by multiple Mann–Whitney tests of two-way ANOVA using the method of two-stage step-up (Benjamini, Krieger, and Yekutieli). A contingency analysis was performed using Chi-square test to compare patients with relapse or not in groups with less and more than 6% of KIR2DL2/3^+^ T cells. Paired t test was used to evaluate whether frequencies of KIR2DL3^+^ versus KIR2DL3^−^ HLA-A2-pp65 specific T lymphocyte degranulation were significantly different. All the statistical tests were done using the GraphPad Prism v6.0 software (San Diego, CA, USA). p values < 0.05 were considered statistically significant.

### Ethics approval

The study complies with the Declaration of Helsinki. In addition, declaration of the preparation and conservation of a bio-collection (DC-2014-2340) was submitted to the French Ministry of Research and had received agreement from the IRB (2015-DC-1) to study lymphocyte reconstitution after transplant. This study was approved by the Ethics Review Board of the Nantes University Hospital and all included patients and blood donors provided informed consent.

## Supplementary Information

Supplementary Information.

## Data Availability

The data that support the findings of this study are included in the article or uploaded as Supplementary Information. Data are available from the corresponding author on reasonable request.

## Abbreviations

HLA: Human leucocyte antigen
KIR: Killer cell immunoglobulin-like receptor
NK: Natural killer
PBMC: Peripheral blood mononuclear cell
