# Supplementary material for: Deciphering the biology of KIR2DL3+ T lymphocytes that are associated to relapse in haploidentical HSCT
Source: Sci Rep. 2021 Aug 4;11:15782. doi: 10.1038/s41598-021-95245-7 (PMC8338934; doi:10.1038/s41598-021-95245-7)
Supplement: Supplementary file 1 — Supplementary Information. [file 41598_2021_95245_MOESM1_ESM.pdf]

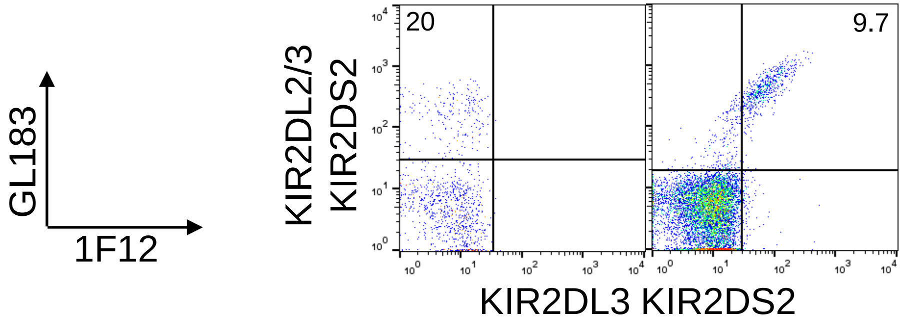

Gene KIR2DS2	+	-
Gene KIR2DL2	+	-
Gene KIR2DL3	-	+

Supplemental figure 1. KIR2DL2 expression on T lymphocytes from the unique patient receiving HSC from KIR2DS2^{+} KIR2DL2^{+} genotyped donor. Density plot illustrating KIR2DL2 expression on T lymphocytes from the unique patient receiving HSC from KIR2DS2^{+} KIR2DL2^{+} genotyped donor. The staining was performed by flow cytometry using the combination of KIR2DL2/3/2DS2 specific GL183 and KIR2DL3/2DS2 specific 1F12mAb leading to identify KIR2DL2^{+} KIR2DL3/2DS2^{-} cells in the upper left gate. The profile of one representative patient receiving KIR2DL3^{+} KIR2DL2/S2^{-} genotyped HSC is shown in parallel with KIR2DL3^{+} cells in the upper right gate. The cell frequency is indicated.
